# Droplet Impact on Asymmetric Hydrophobic Microstructures

**DOI:** 10.1021/acs.langmuir.2c00561

**Published:** 2022-06-23

**Authors:** Susumu Yada, Ugis Lacis, Wouter van der Wijngaart, Fredrik Lundell, Gustav Amberg, Shervin Bagheri

**Affiliations:** † FLOW Centre, Department of Engineering Mechanics, Royal Institute of Technology (KTH), 100 44 Stockholm, Sweden; ‡ Division of Micro and Nanosystems, Royal Institute of Technology (KTH), 100 44 Stockholm, Sweden; § Södertörn University, 141 89 Stockholm, Sweden

## Abstract

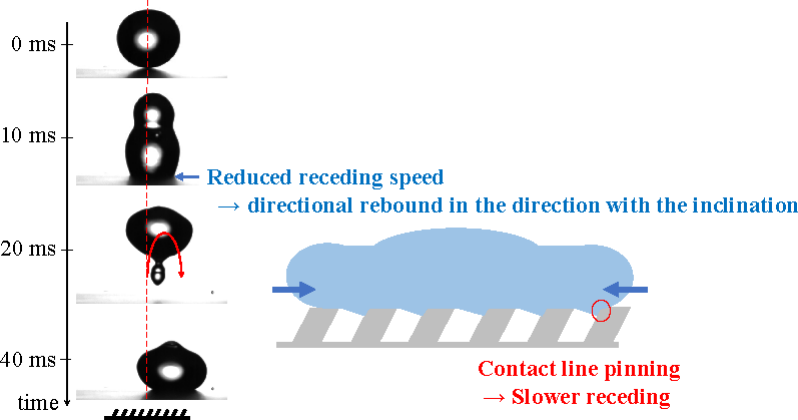

Textured hydrophobic surfaces that repel liquid droplets unidirectionally are found in nature such as butterfly wings and ryegrass leaves and are also essential in technological processes such as self-cleaning and anti-icing. In many occasions, surface textures are oriented to direct rebounding droplets. Surface macrostructures (>100 μm) have often been explored to induce directional rebound. However, the influence of impact speed and detailed surface geometry on rebound is vaguely understood, particularly for small microstructures. Here, we study, using a high-speed camera, droplet impact on surfaces with inclined micropillars. We observed directional rebound at high impact speeds on surfaces with dense arrays of pillars. We attribute this asymmetry to the difference in wetting behavior of the structure sidewalls, causing slower retraction of the contact line in the direction against the inclination compared to with the inclination. The experimental observations are complemented with numerical simulations to elucidate the detailed movement of the drops over the pillars. These insights improve our understanding of droplet impact on hydrophobic microstructures and may be useful for designing structured surfaces for controlling droplet mobility.

## Introduction

Droplet deposition and impact are important in applications such as spray coating and cooling,^1,2^ pesticide deposition,^3,4^ and inkjet printing.^5,6^ They are also relevant for emerging applications such as electricity generation using droplets^7^ and efficient thermal cooling.^8^ Droplet impact involves complex fluid motions including splashing,^9−13^ the formation of a thin gas layer between the droplet and the surface,^14−17^ and droplet rebound on superhydrophobic surfaces.^18−22^ Theoretical,^23−26^ numerical,^25−28^ and experimental investigations^20,29−35^ of droplet impact have also highlighted the fingering of spreading front and the scaling laws for maximum deformation.^2,36,37^ Specific features of droplet rebound such as symmetry break of droplet impact on a curved surface^38^ and on macrotextures^39^ have also received attention.

Droplet rebound dynamics on hydrophobic microstructured surfaces are generally discussed in terms of the stability of wetting states. Microstructured surfaces trap air underneath droplets (i.e., the Cassie wetting state), rendering the surface significantly more hydrophobic. The robustness of the air cavity and the resulting droplet behavior has been studied in terms of impalement pressure.^18,19,22,40^ When the fluid pressure exceeds a critical pressure on a structured surface, the Cassie–Wenzel wetting transition occurs and droplets cease to rebound.

Asymmetric hydrophobic microstructures are often exploited by natural species, such as butterfly wings^41^ and ryegrass leaves^42^ where they assist liquid roll-off. Surfaces with asymmetric ratchets and spikes allow directing a droplet in a desired direction, and such anisotropic surfaces are useful particularly in self-cleaning, water harvesting,^43^ and cell directing.^42,44,45^ Here, hydrophobic surface properties are advantageous to increase the mobility of a droplet. On such hydrophobic surfaces, upon impact, droplets bounce off toward the direction in which the surface structures are oriented.^41,46−51^

Wang et al.^46^ first achieved droplet rebound in a guided direction on inclined macrostructures (300 μm tall needles). Since then, droplet rebound on asymmetric structures has been explored with different surface fabrication techniques such as soft lithography,^48,49^ molding,^50^ and 3D printing^51^ and for different applications. Therefore, surface parameters such as height, spacing, and shape are broad and are critical for rebound behaviors. For example, Tao et al. reported that inclined Janus (half-cone with a flat sidewall) structures are more advantageous for directional droplet rebound than conical inclined structures.^51^ Particularly, the influence of the length scale of such structures is of interest. Directional pancake bouncing without retraction has been observed for hairy macrostructures (>100 μm).^47,50−52^ However, smaller asymmetric microstructures (∼10 μm) have not been well investigated. Moreover, investigation of the influence of the impact speed is limited. The influence of the surface geometry, that is, the pitch and height of structural features, and the impact velocity remain to be fully elucidated.

Here, we study droplet impact on asymmetric microstructures experimentally and numerically. We note that the ∼10 μm microstructures in this study are 1 or 2 orders of magnitude smaller than those in most previous studies,^41,47−51,53^ which were on the order of 100–1000 μm. The dimension is also smaller than the capillary length , where ρ and σ are the density and surface tension of water and *g* is the gravitational acceleration. We observe a distinct influence of surface geometry and impact velocity on impact behavior. Moreover, we measure the trajectories of bouncing droplets and investigate the conditions for directional rebound. We observe and discuss differences in receding speeds of the contact line in the direction with the inclination and against the inclination.

## Materials and Methods

### Experimental Setup

**Figure 1:**
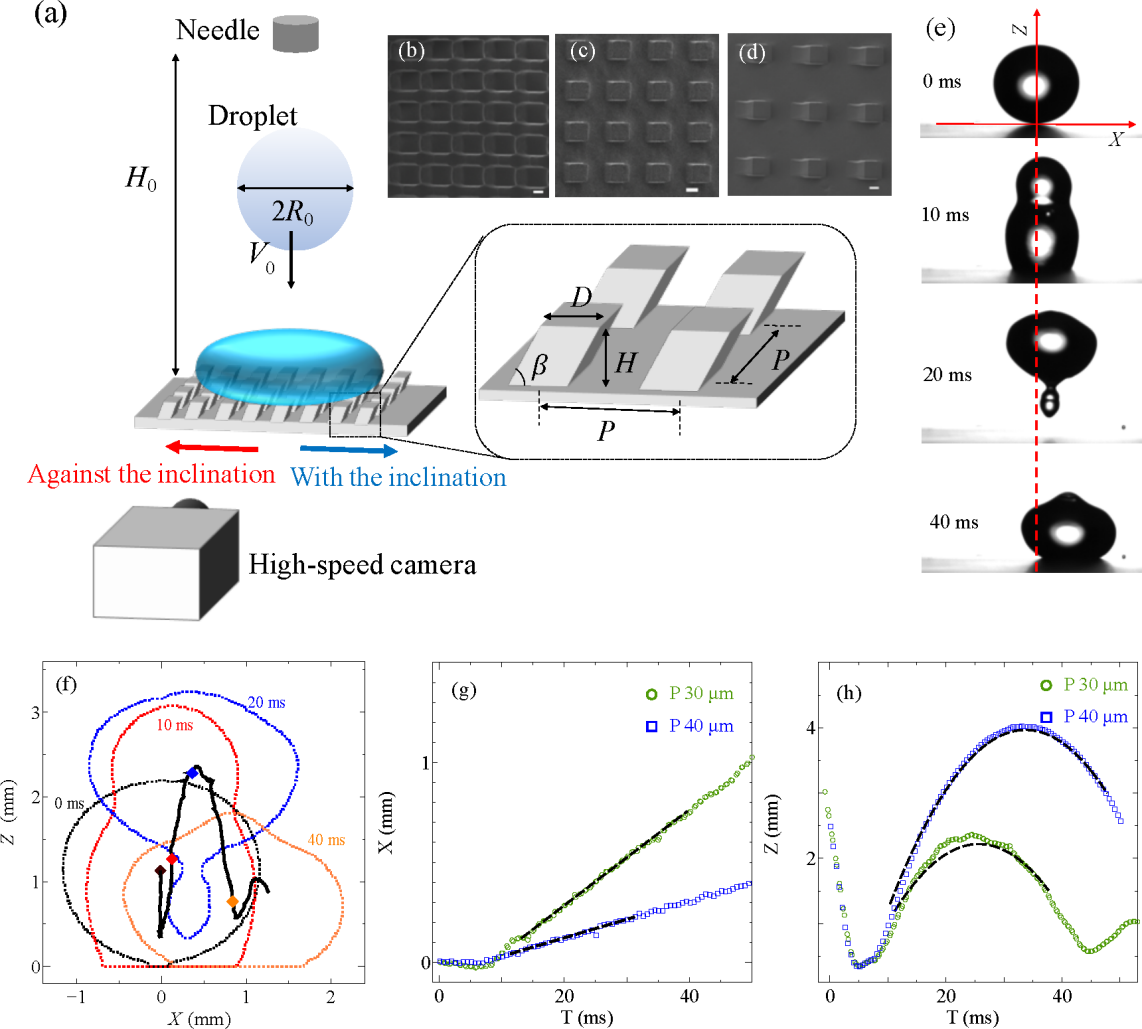
(a) Schematic description of the droplet impact experiment. (b–d) Scanning electron microscopy image of the inclined microstructure with (b) *P* = 30 μm, (c) *P* = 40 μm, and (d) *P* = 60 μm. The scale bar indicates 10 μm. (e) Selected snapshots from experiments (*P* = 30 μm and *V*_0_ = 0.56 m/s). The surface structures are inclined to the right. (f–h) Procedure to estimate rebound velocity. (f) Captured droplet shape (dotted lines) and the trajectory of center of mass (black solid line) from (e). The positive *X* indicates the horizontal direction with the inclination of the pillars, and *Z* is the vertical displacement from the substrate. (g, h) Horizontal and vertical position of the center of mass as a function of time. Dashed black lines in (g, h) are ballistic trajectories (eqs 1 and 2) with fitted *V*_*x*_ and *V*_*z*_. A typical trajectory for *P* = 40 μm and *V*_0_ = 0.56 m/s is also shown in (g, h).

The impact of liquid droplets is observed with a high-speed camera (SpeedSense, Dantec Dynamics) at a frame rate of 6000–8000 s^–1^ with a spatial resolution of 15 μm. The schematics of the experimental setup are shown in Figure 1a. A liquid droplet is formed on the tip of a needle with an outer diameter of 0.31 mm (Hamilton, gauge 30, point style 3) at a height *H*_0_ from the surfaces. The liquid is pumped by a syringe pump (Cetoni, neMESYS 1000N) at a small flow rate (0.10 μL/s), and the droplet pinches off from the needle with the constant initial radius *R*_0_ = 1.14 ± 0.02 mm. The droplet is accelerated by gravity and hits the substrate with an impact velocity *V*_0_. The impact velocities are varied by changing the distance from the substrate to the needle *H*_0_. The impact velocity is estimated from the images just before the droplet hits the substrate. The captured images are shown in Figure 1e. The height *H*_0_ is varied from 5 to 85 mm, which leads to impact velocities *V*_0_ from 0.25 to 1.3 m/s (Table 1).

**Table 1:** List of the Heights *H*_0_, the Impact Velocities *V*_0_, and Weber Number *We* = ρ*R*_0_*V*_0_^2^/σ

*H*_0_ (mm)	5	10	15	20	25	40	60	85
*V*_0_ (m/s)	0.25	0.38	0.50	0.56	0.64	0.84	1.1	1.3
*We*	0.9	2.3	3.8	5.1	7.3	10.8	16.8	25.8

The liquid employed in this study is deionized water. The surface tension of water σ is measured to be 0.072 mN/m with a TD 2 tensiometer (LAUDA). In this study, we focus on the droplet motion in the direction of the inclination of the pillars. Droplet dynamics in the direction orthogonal to the inclination is expected to be similar to straight pillars. Spreading and retraction are expected to be symmetric, and therefore, the rebound is expected to be straight up.^22^

### Surface Preparation

The substrates studied are made from Ostemer 220 (Mercene Labs AB, Sweden), Off-Stoichiometry-Thiol–Ene (OSTE) resin.^54,55^ The resin is suitable for fabricating inclined micropatterns by exposing slanted collimated ultraviolet light. The surfaces are prepared in three steps. First, a base OSTE layer is prepared on a smooth plastic film. Second, inclined micropillars are developed on the base layer by exposing slanted ultraviolet light through a patterned photomask. After cleaning uncured OSTE in an acetone bath, hydrophobic surface modification using 1% w/w fluorinated methacrylate (3,3,4,4,5,5,6,6,7,7,8,8,9,9,10,10,10-heptadecafluorodecyl methacrylate, Sigma-Aldrich) solution in 2-propanol with 0.05% benzophenone (Sigma-Aldrich) initiator is applied. Surface structures are characterized with scanning electron microscopy (see Figure 1b–d), and the inclination of the pillars β is 60°.

The equilibrium contact angles of deionized water are reported in Table. 2. The advancing and receding contact angles are measured by using the sessile drop method.^56,57^ A droplet with the initial volume of 5 μL is deposited on the surface, and it is pumped through the needle at a flow rate of 0.1 μL/s to measure advancing contact angle. For the receding angle measurements, the initial volume is set to 30 μL to perform reliable measurements,^57^ and the droplet is drained at a flow rate of 0.1 μL/s. The average contact angle for 5 s after the contact line starts to move is defined as the advancing (receding) contact angle.

**Table 2:** List of the Surfaces^a^

	*D* (μm)	*P* (μm)	*H* (μm)	θ_e_ (deg)	θ_a–A_ (deg)	θ_a–W_ (deg)	θ_r–A_ (deg)	θ_r–W_ (deg)
flat				112 ± 2	121 ± 3		73 ± 3	
P30	20	30	20	133 ± 3	150 ± 2	146 ± 4	84 ± 8	100 ± 4
P40	20	40	20	146 ± 1	147 ± 6	147 ± 5	115 ± 5	117 ± 5
P60	20	60	20	109 ± 3	112 ± 5	117 ± 5	62 ± 5	58 ± 5

a *D*, *P*, and *H* are the width, pitch, and height of the pillars. θ_e_ is the equilibrium contact angle. θ_a–A_, θ_a–W_, θ_r–A_, and θ_r–W_ are advancing/receding contact angle in the direction against the inclination and with the inclination, respectively. The advancing/receding contact angles in the direction against the inclination and with the inclination on the flat surface are identical.

### Rebound Velocity Estimation

To investigate the influence of the surface structure and the impact velocity on rebound behaviors, the trajectory of the droplet is calculated. The trajectory of the center of mass is obtained by extracting the surface contour from the images (Figure 1e,f). Assuming ballistic trajectory after the impact, the horizontal and vertical positions *X* and *Z* are described as a function of time *T*
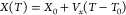
1
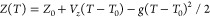
2where the positive *X* indicates the horizontal direction with the inclination of the pillars and *Z* is the vertical displacement from the substrate. Here, *V*_*x*_ and *V*_*z*_ are horizontal and vertical velocities, *g* is the gravitational acceleration, and *X*_0_ and *Z*_0_ are the horizontal and vertical positions at *T* = *T*_0_. Equations 1 and 2 describe the trajectory well when *V*_*x*_ and *V*_*z*_ are fitted (see the dashed lines in Figure 1g,h). By performing the fitting procedure, we estimated *V*_*x*_ and *V*_*z*_ as a function of the impact velocity. Here, *X*_0_ and *T*_0_ are set so that *Z*_0_ = 1.1*R*_0_ for all configurations.

## Results and Discussion

### Bouncing Regimes

**Figure 2:**
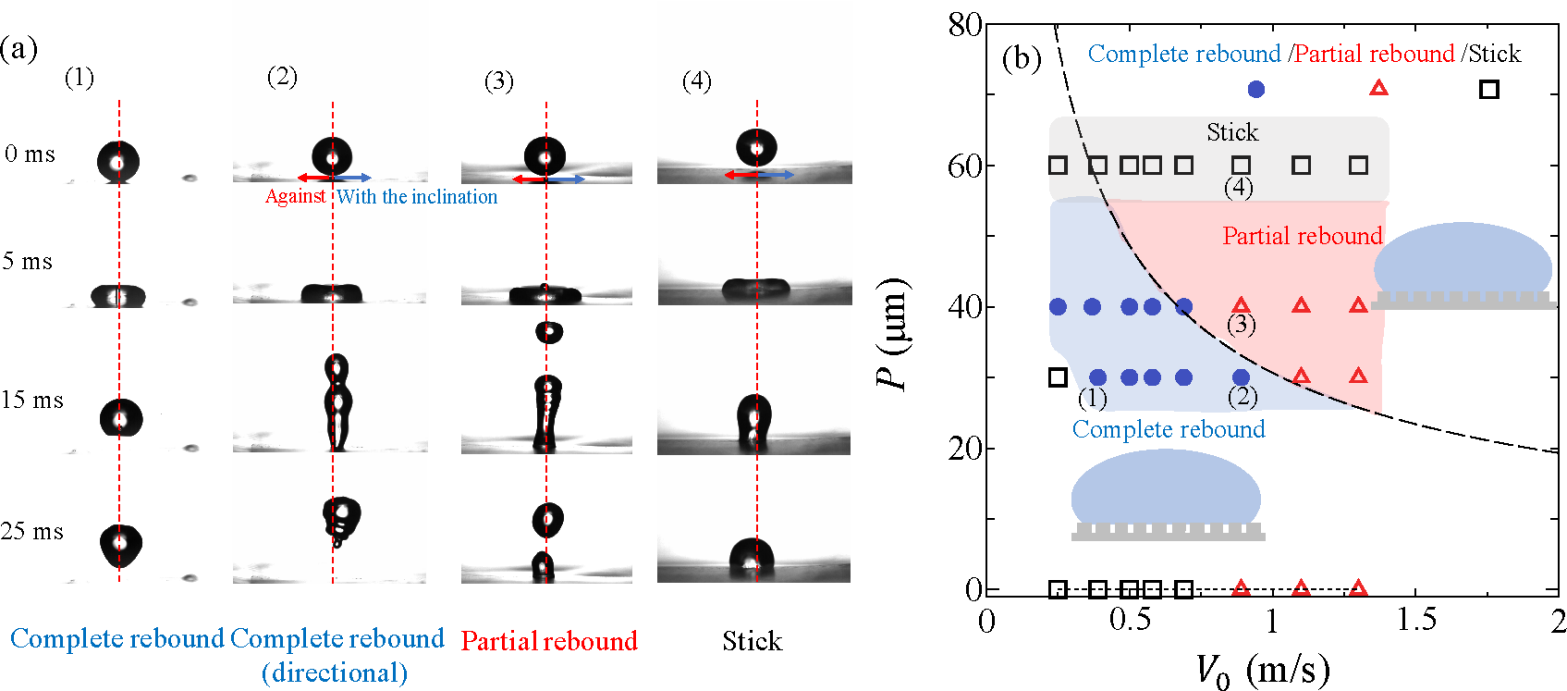
(a) Selected snapshots from the experiments. (1) *V*_0_ = 0.38 m/s on *P* = 30 μm, (2) *V*_0_ = 0.84 m/s on *P* = 30 μm, (3) *V*_0_ = 0.84 m/s on *P* = 40 μm, and (4) *V*_0_ = 0.84 m/s on *P* = 60 μm. The surface structures are inclined to the right. (b) Impact velocity-pitch map for different behavior after droplet impact. Blue, red, and black marks indicate complete rebound, partial rebound, and stick behavior, respectively. *P* = 0 indicates the flat surfaces. The dashed curve describes the semiquantitative model for an array of straight pillars by Bartolo et al.^18^. The inset shows the schematic for the wetting transition. The numbers 1–4 correspond to the snapshots in (a).

Figure 2 shows a series of images of a water droplet spreading after impact. We observe that the pitch between the pillars *P* and the impact velocity *V*_0_ determine the droplet behavior. Three distinctive behaviors are observed. First, the droplet completely rebounds from the surface (1 and 2 in Figure 2a). Moreover, the droplet rebounds to the direction with the inclination on *P* = 30 μm and at high *V*_0_ (case 2 in Figure 2a). Second, the droplet breaks up, and part of the droplet remains deposited on the surface while the other part bounces up (3 in Figure 2a). We note that the directional move of the secondary droplet is small. Finally, the droplet does not bounce and sticks to the surface (4 in Figure 2a). We refer to the three configurations as “complete rebound”, “partial rebound”, and “stick”. Figure 2b shows the pitch-impact velocity parameter map with the “complete rebound”, “partial rebound”, and “stick” regions indicated.

The Cassie–Wenzel transition is responsible for the different behaviors. For “complete rebound” situations, the grooves between the posts are not wetted, and air is trapped underneath the droplet (Cassie state). On the other hand, for “partial rebound” and “stick” cases, the grooves are partially or fully penetrated by the liquid (Wenzel state). A semiquantitative model to account for the Cassie to Wenzel transition on an array of pillars was proposed by Bartolo et al.^18^ The model estimates the critical impalement pressure on a structured surface. When the hydrodynamic pressure over the surface exceeds the critical pressure, the liquid–air interface makes contact with the basal surface of the substrate, and the liquid penetrates into the grooves. Above the critical pressure, the Cassie–Wenzel wetting transition occurs, which also corresponds to the transition from bouncing to nonbouncing. The model estimates the critical pressure as *p*_c_ ∼ σ*HD*/2*P*^3^ for dense arrays of straight pillars,^18^ where *H* and *D* are the height and width of the pillars, respectively. In the instant of droplet impact, the hydrodynamic pressure is *p*_d_ ∼ ρ*V*_0_^2^/2, where ρ is the density of the liquid. The balance *p*_c_ ∼ *p*_d_ gives the critical impact velocity for the pitch *P* as . The dashed curve in Figure 2b depicts this critical value. We observe that the critical curve separates the complete rebound regime and partial rebound regime reasonably well also for inclined pillars. Beyond the critical impact velocity, “stick” and “partial rebound” are observed.

### Rebound Velocity

As seen in the previous sections, the rebound behavior in the horizontal direction depends on the surface structure and the impact velocity. This section investigates the directional behavior within the rebound regime.

**Figure 3:**
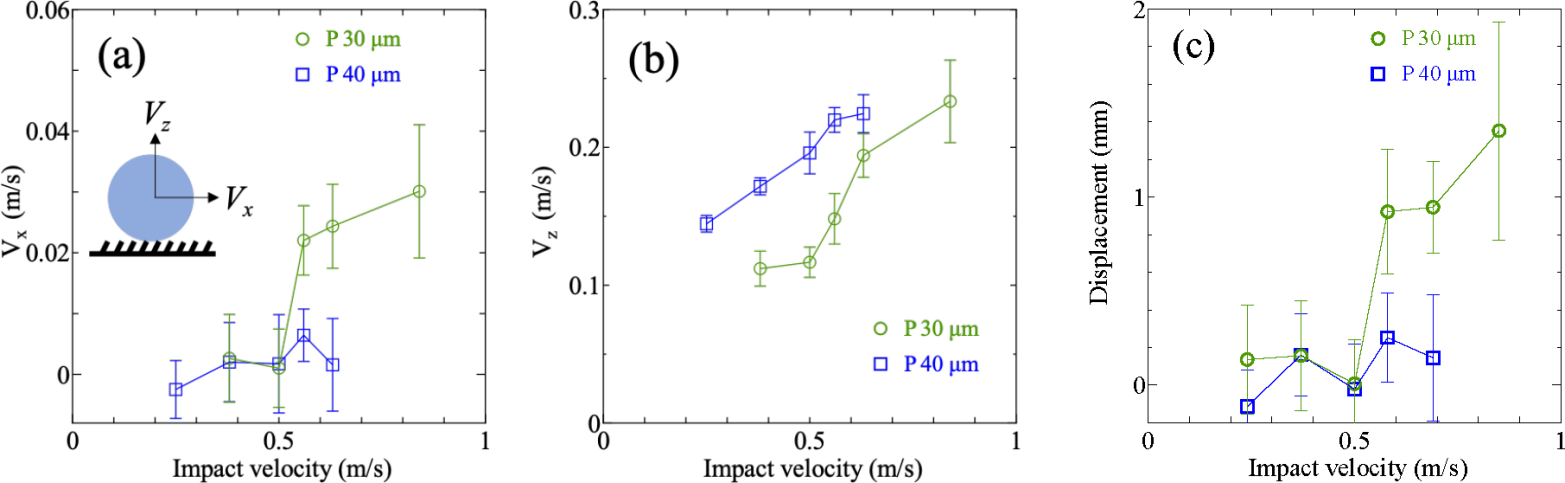
Influence of impact velocity on droplet rebound velocity. (a) Horizontal rebound velocity *V*_*x*_. (b) Vertical rebound velocity *V*_*z*_. (c) Terminal horizontal displacement for different impact velocity. Error bars indicate standard deviations. The data are averages over more than eight separate measurements.

Figure 3a shows *V*_*x*_ at different impact velocities. The horizontal velocity *V*_*x*_ is negligibly small for low impact velocity (<0.5 m/s) and increases to ∼0.03 m/s with the impact velocity for *P* = 30 μm. For *P* = 40 μm, *V*_*x*_ remains small even for the highest impact speed.

The vertical rebound velocity *V*_*z*_ in Figure 3b increases with the impact velocity up to ∼0.25 m/s. Larger *V*_*z*_ is observed for *P* = 40 μm compared to *P* = 30 μm. This is likely because of the higher level of hydrophobicity, which is indicated by the larger equilibrium contact angle on *P* = 40 μm (see Table. 2). The droplet moves in the direction with the inclination up to 1.3 mm (Figure 3c). The directional displacement is observed only for *V*_0_ > 0.5 m/s and on *P* = 30 μm. This is similar to the observation made by Li et al.^49^ They also observed a larger horizontal displacement on arrays of inclined cones with a smaller spacing.

**Figure 4:**
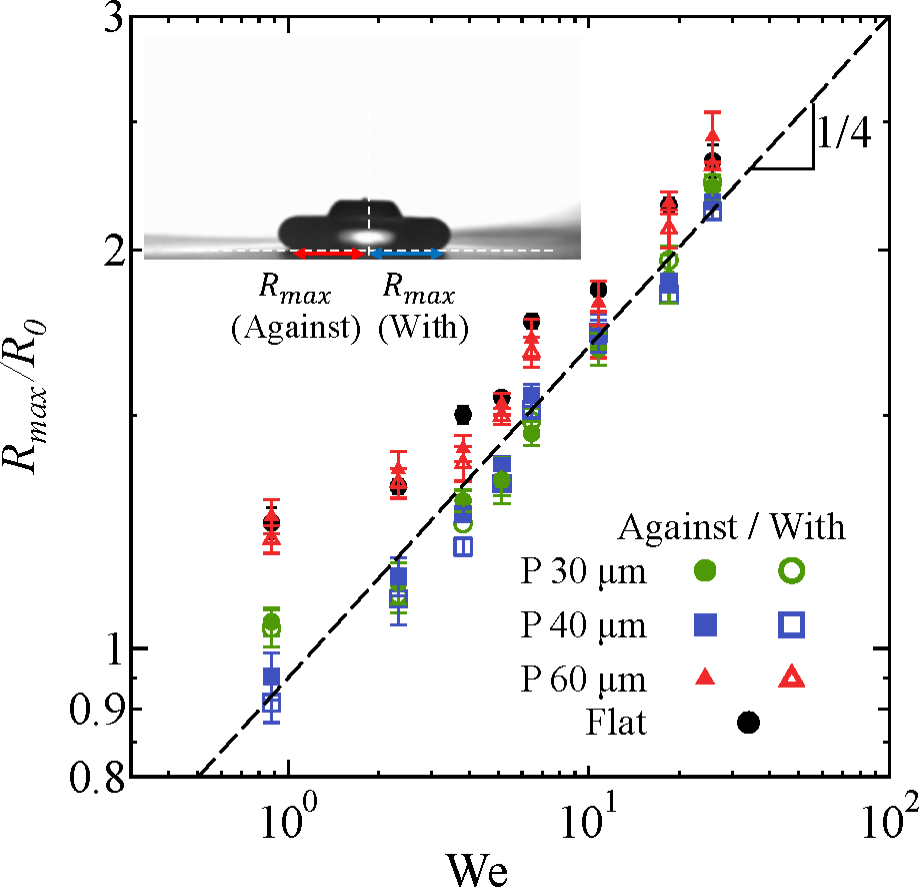
Normalized maximum contact radius as a function of Weber number *We* = ρ*R*_0_*V*_0_^2^/σ. The dashed line indicates *R*_max_ ∝ *We*^1/4^. The inset describes the definition of *R*_max_. The surface in the inset is oriented to the right.

It is noticeable that the expansion phase until the droplet reaches the maximum deformation is symmetric on the inclined hydrophobic pillars (see the snapshots at 5 ms in Figure 2). This is further quantified in Figure 4, where the maximum contact radius of the droplet *R*_max_/*R*_0_ is shown as a function of the Weber number *We* = ρ*R*_0_*V*_0_^2^/σ. The maximum contact radii in the direction with the inclination and against the inclination are similar for all surfaces. The maximum contact radius for *P* = 30 μm and *P* = 40 μm are slightly smaller than for the flat surface, while it is nearly the same as the flat surface for *P* = 60 μm. Furthermore, the maximum contact radius follows the well-known relation^29,33^*R*_max_ ∝ *We*^1/4^. This is consistent with previous studies with low Ohnesorge number , where μ is the liquid viscosity, while more viscous fluids exhibit a smaller exponent (∼1/6).^24,29,33^ The Ohnesorge number in this study is 3.5 × 10^–3^, which is reasonably low.

Contrary to the first expansion, the retraction immediately after the initial expansion can be asymmetric. The asymmetric retraction is responsible for the observed asymmetric bouncing. The retraction is governed by how the contact line detaches from the surface structures.

**Figure 5:**
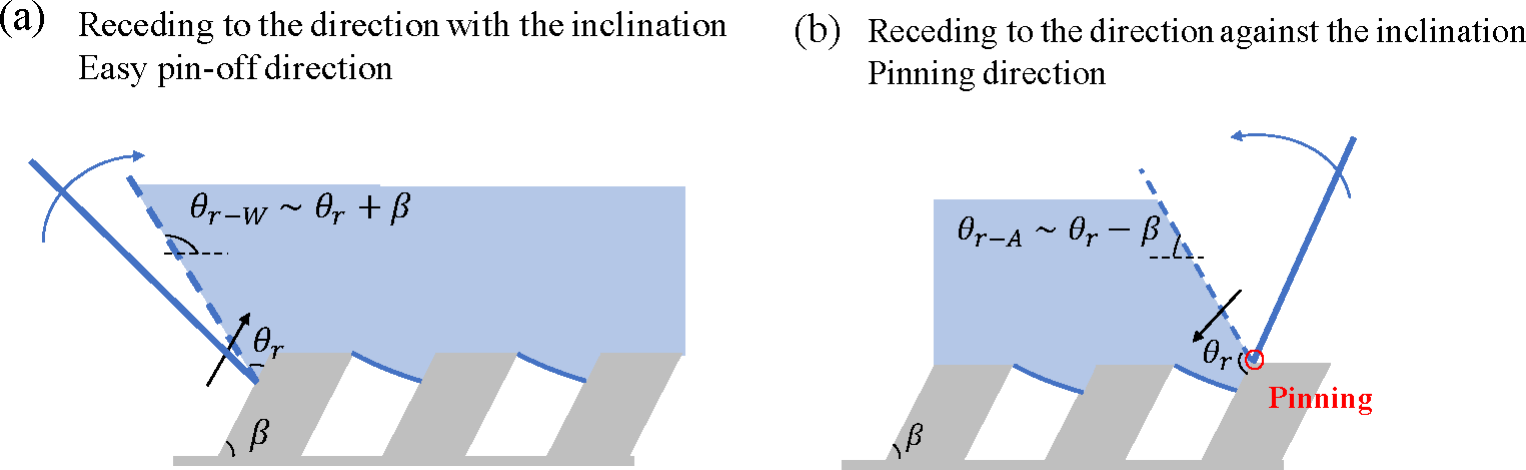
Schematic models of the receding contact lines (a) in the direction with the inclination and (b) in the direction against the inclination.

The underlying mechanism of the asymmetric receding speed is in the wetting of the asymmetric microstructure. Figure 5 shows the schematic model of the receding contact line on the asymmetric microstructure. The key factor is that a part of the inclined sidewall is wetted. The wetting on asymmetric microstructured surfaces was also illustrated by Guo et al.^42^ and Malvadkar et al.^44^ for rolling-off droplets.

The contact line recedes when the local contact angle decreases to the intrinsic receding contact angle, θ_r_ = 73°. When the contact line recedes in the direction with the inclination on the inclined wall (from left to right in Figure 5a), the apparent receding angle θ_r–W_ is θ_r_ + β ∼ 133°. Therefore, the contact line smoothly recedes on the sidewall. On the other hand, the apparent receding angle in the direction against the inclination (θ_r–W_)—when the contact line moves down on the sidewall—should be θ_r_ – β, i.e. as small as 13° (Figure 5b). The contact line is then pinned at the obtuse corner until the liquid detaches from the sidewall. This pinning delays the receding in the direction against the inclination. Note that the liquid inertia helps the interface detach from the sidewall, so the apparent receding angle in the experiments is not as small as θ_r_ – β.

### Numerical Simulations of Droplet Impact

The mechanism described above can be confirmed with numerical simulations of droplet impact. The simulations are used to qualitatively reveal the spreading and receding mechanisms on the asymmetric microstructures, which cannot be resolved with our experimental setup. The droplet impact on the asymmetric microstructure is modeled with Navier–Stokes–Cahn–Hilliard equations. The Cahn–Hilliard equation describes the time evolution of the two-phase system based on the diffusion of the chemical potential of the system, whereas the Navier–Stokes equations describe the incompressible flow field. The simulations are performed in a two-dimensional geometry to keep the computational cost feasible. Therefore, we limit the use of the simulations to investigate the contact line behaviors qualitatively in a two-dimensional view. The droplet radius and the impact velocity are set to 0.125 mm and 1–2 m/s, respectively. Note that the impact velocity, the relative scale of the microstructures to the droplet, and the surface geometry are different from the experiments. The radius of the droplet is reduced from the experiments to keep computational cost feasible, and the impact speed is increased to induce sufficient deformation and retraction. The details of the simulations are provided in the Supporting Information.

**Figure 6:**
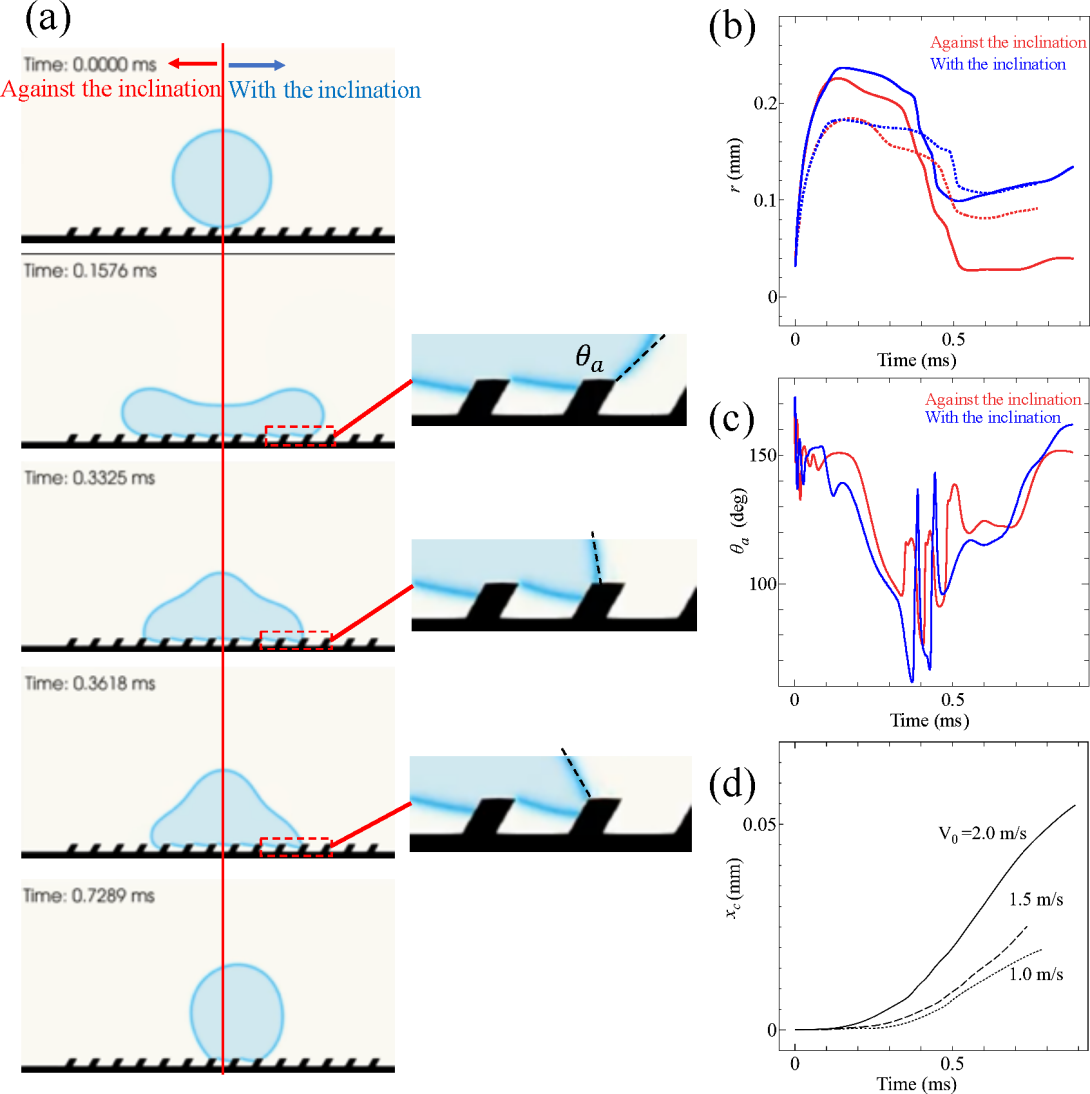
Simulations of a droplet impacting on an asymmetric hydrophobic microstructure. (a) Selected pictures from the simulation. The inset provides a magnified picture near the contact line. The impact velocity in (a) is 2 m/s. (b) Contact radii from the initial center of the droplet. The solid and dotted lines in (b) indicate *V*_0_ = 2 and 1 m/s, respectively. (c) Apparent contact angle θ_a_. The impact velocity in (c) is 2 m/s. (d) Center of mass *x*_c_. A positive *x*_*c*_ indicates the horizontal direction with the inclination. The solid, dashed, and dotted lines in (d) indicate *V*_0_ = 2, 1.5, and 1 m/s, respectively.

Figure 6a shows snapshots from the simulation of a droplet impacting on the inclined microstructures with *V*_0_ = 2 m/s. The droplet is displaced in the direction with the inclination, although the droplet does not detach from the surface. The meniscus between the pillars is maintained as in the inset of Figure 6a, and then the wetting mechanisms would be similar to the “complete rebound” situation, even though the droplet does not rebound from the surface. The droplet does not bounce off since the two-dimensional surfaces in the simulations are not hydrophobic enough. Here, the sidewalls of the inclined structures are wetted during the spreading (see the inset in Figure 6a). The spreading is nearly symmetric until 0.2 ms, and then the contact line starts to recede (see Figure 6b). During the retraction in the direction with the inclination, the liquid is arrested on the sidewall of the structures. While the contact line is pinned at the obtuse corner, the liquid phase cannot detach from the sidewall (see the inset in Figure 6a). The apparent contact angle has to decrease below 60° before the contact line detaches from the sidewall (see Figure 6c). On the other hand, during the retraction in the direction against the inclination, the apparent contact angle does not become lower than 80°. Consequently, the retraction is faster in the direction against the inclination than in the direction with the inclination. This difference is responsible for the directional motion in the direction with the inclination.

Moreover, the numerical simulations with different impact velocities are consistent with our experimental observation. Figure 6d shows the horizontal displacement of the center of the mass of the droplet with different impact velocities. The larger the impact speed, the faster the horizontal motion becomes. As seen in Figure 6b, the retraction distance is larger for a higher impact velocity. Here, the longer retraction distance of the contact line is responsible for the stronger effect of the pinning, which leads to the larger displacement.

### Receding Contact Angle Measurements

**Figure 7:**
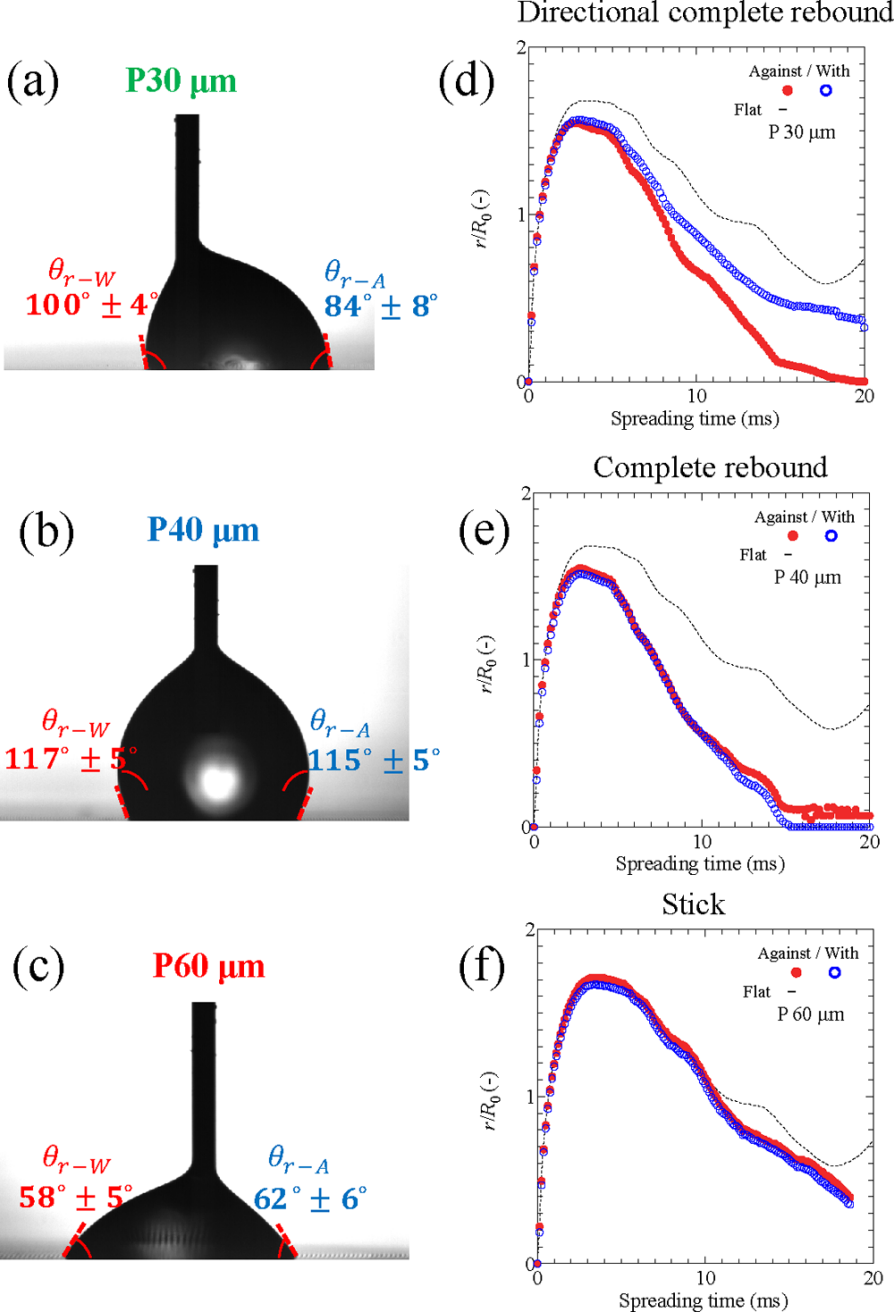
(a–c) Measured receding contact angles on the asymmetric microstructures. The pictures show the typical droplet shapes after the contact line starts to recede. (d–f) Corresponding droplet impact behavior. Contact line position from the initial center of the droplet for the impact speed *V*_0_ = 0.64 m/s (*H*_0_ = 25 mm). The droplet rebounds directionally in the direction with the inclination on *P* = 30 μm, rebounds vertically on *P* = 40 μm, and sticks on *P* = 60 μm.

Here, we demonstrate that the receding contact angle measured with the sessile drop method is consistent with the droplet impact behavior. The receding contact angle in the direction against the inclination (θ_r–A_ = 84°) is smaller than in the direction with the inclination (θ_r–W_ = 100°) for *P* = 30 μm (Figure 7a). This is consistent with the fact that the receding speed in the direction against the inclination during the droplet impact is slower (Figure 7d). This difference leads to directional rebound. Meanwhile, the receding angles on the surfaces with *P* = 40 and 60 μm are similar for the two directions (Figure 7b,c). The symmetry in the receding contact angle is consistent with the symmetric receding speed during the droplet impact (Figure 7e,f).

The pitch in the direction perpendicular to the direction of the inclination of the surface structures is potentially responsible for the difference between *P* = 30 μm and *P* = 40 μm where the droplet rebounds in both cases. The effect of the pinning described in Figure 5 is effective only when the pillars are sufficiently dense along the contact line so as for the pinning to be effective enough to delay the receding. This implies that the pinning site is dense enough only for *P* = 30 μm but not for *P* = 40 μm in our experiments. Moreover, the mechanisms in Figure 5 are undermined for “partial rebound” and “stick” cases since the grooves between the posts are filled with water. Therefore, the directional behavior is not expected for “partial rebound” and “stick” droplets.

### Discussion

The mechanisms underlying asymmetric droplet rebound elucidate the influence of the surface geometries on rebound behavior. To realize a directional rebound on arrays of inclined micropillars, three conditions must be fulfilled. First, the receding contact line speeds in the directions with and against the inclination need to be different. Second, the grooves between the pillars should not be completely wetted. This condition corresponds to the complete rebound regime in the Cassie–Wenzel transition model. Third, the impact velocity should be large enough to deform the droplet and lead to substantial retraction. To satisfy these three conditions, a surface should have both sufficient pinning corners in the direction perpendicular to the inclination of the surface structures and be hydrophobic enough to induce a rebound.

**Figure 8:**
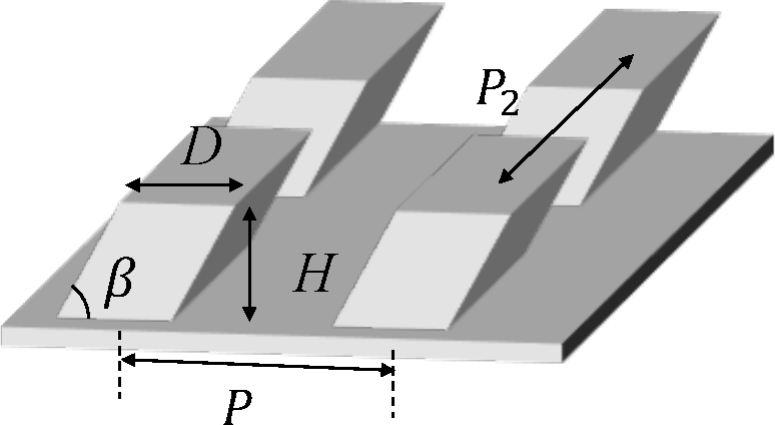
Another pitch in the direction perpendicular to the inclination of the surface structures, *P*_2_.

There is, however, a trade-off between the density of the pinning corners and the hydrophobicity of the surface. As the pitch decreases (*P* → 0), the number of pinning sites along the contact line increases, but the surface becomes less hydrophobic, as the solid–air ratio increases. An additional degree of freedom of the surface to enhance the directional rebound is the pitch *P*_2_ in the direction perpendicular to the direction of the inclination (see Figure 8). Because it is desirable to increase the number of pinning sites while keeping the surface hydrophobic, structures with *P* > *P*_2_ and a reasonably large static contact angle could enhance the directional rebound.

**Figure 9:**
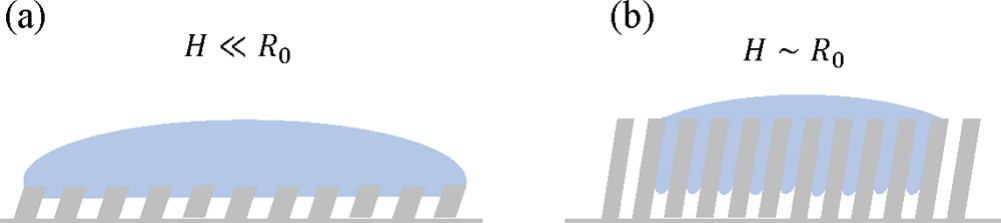
Schematic description of the wetting and rebound scenario on microstructures (a) with small height compared to the radius of the droplet and (b) with comparable height to the radius of the droplet.

The directional rebound mechanisms proposed in previous studies are different in certain aspects than in this study. Lee et al.^47^ proposed that the stored surface energy between the inclined structures is responsible for the directional rebound. Note that the height of their structures is on the order of 1 mm, which is 2 orders of magnitude higher than in this study (and the aspect ratio of the surface dimension is large). For such surfaces, the droplet can fully penetrate the pillar arrays during impact. A similar large penetration was observed by Li et al.^48^ The two different situations are schematically shown in Figure 9. Lee et al.^47^ and Liu et al.^58^ express the change in surface energy during the penetration as *E*_s_ ∼ 4σ*n*_p_*h*^2^|cos θ_e_|, where *n*_p_ is the number of wetted pillars and *h* is the penetration depth. The surface energy is expected to transform to the kinetic energy of the bouncing droplet, assuming viscous dissipation is negligible. The eject velocity given by the surface energy is directed into the direction of the inclination, led by capillary forces. Assuming *n*_p_ ∼ (*R*_0_/*P*)^2^ and *h* ∼ *H*, *E*_s_ ∼ 4σ*R*_0_^2^(*H*/*P*)^2^|cos θ_e_|. Because *E*_s_ is proportional to the square of the ratio of the height and the pitch *H*/*P*, the change of surface energy by the penetration could be small for our surface. For example, for an order of magnitude estimation, for a droplet with an initial radius of 1 mm, *E*_s_ ∼ *O*(10^–7^) for our structures with *H*/*P* ∼ 1 while *E*_s_ ∼ *O*(10^–5^) for Lee et al. with *H*/*P* ∼ 10.^47^ This is equal to the kinetic energy of the droplet with velocity ∼0.1 m/s for our structures and ∼1 m/s for Lee et al. Therefore, the change of surface energy from the penetration is insignificant for surface roughness with an aspect ratio of ∼1 and *H* ≪ *R*_0_. Instead, the difference in the receding contact line speed is responsible for the directional rebound.

The different mechanisms may explain the differences in the horizontal velocity and the displacement. Here, we compare the horizontal velocity and displacement distance on the inclined microstructures with β ∼ 60° in the literature. For a smaller structure where the rebound mechanisms are described in Figure 9a, smaller displacement and velocity are observed. Li et al.^49^ reported that on inclined cone structures with *H* ∼ 300 μm and *P* ∼ 300–400 μm the displacement is 0.7 mm for *V*_0_ = 0.71 m/s and 2.0 mm for *V*_0_ = 1.73 m/s. The horizontal rebound distance is similar to this study.

On the other hand, for a very tall structure with *H* > 1 mm and *P* < 500 μm, where the rebound may follow the scenario in Figure 9b, a larger rebound velocity is observed. Lee et al.^47^ reported the horizontal velocity and displacement on thin-spike structures. The horizontal velocity is 0.09 m/s, and the displacement distance is 9.1 mm for *V*_0_ = 1.13 m/s. Similarly, Li et al.^48^ reported a large horizontal velocity and horizontal displacement of 0.062 m/s and 5.5 mm, but the impact velocity information is missing. The difference in the mechanisms could be responsible for the larger displacement since the surfaces described in Figure 9b are capable of harnessing droplets with larger impact speed and therefore able to store larger surface energy before the rebound. It is worth noting that Lee et al.^47^ also pointed out that the bouncing mechanisms on superhydrophobic nanostructures and hairy spike structures are different. While the droplet rebounds after the full retraction on the superhydrophobic nanostructures, the droplet rebounds by upward capillary forces on their hairy spike arrays.

## Conclusions

We studied the droplet impact on asymmetric microstructures. Directional rebound was observed only for dense microstructures and at high impact speeds. The retraction phase and the detailed wetting of the sidewalls of the inclined structures govern the rebound. The wetting of the sidewall leads to a slower receding speed in the direction against the inclination. The contact line can be pinned at the obtuse corner when receding in the direction against the inclination, while in the other direction the contact line recedes continuously. The receding contact angles on the asymmetric pillar structures correlate with the droplet rebound behavior. The directional rebound is found only on surfaces with asymmetric receding contact angles in the direction of the pillar inclination. Numerical simulations provide further detailed visualizations of the two phase interface near the pillars and confirm our experimental observations. We hope that insights gained in this study will be useful for tuning surface structures for directional transport of liquid drops.

## Supporting Information Available

Detailed numerical methods, including the computational domain (PDF)
Video animations of a simulated droplet impact (MP4)
