# Supplementary material for: Droplet Impact on Asymmetric Hydrophobic Microstructures
Source: Langmuir. 2022 Jun 23;38(26):7956–64. doi: 10.1021/acs.langmuir.2c00561 (PMC9261186; doi:10.1021/acs.langmuir.2c00561)
Supplement: Supplementary file 1 — la2c00561_si_001.pdf [file la2c00561_si_001.pdf]

Supporting Information: Droplet impact on asymmetric hydrophobic microstructures

Susumu Yada,[†] Ugis Lacis,[†] Wouter van der Wijngaart,[‡] Fredrik Lundell,[†] Gustav Amberg,[†,¶] and Shervin Bagheri[*,†]

†*FLOW Centre, Dept. of Engineering Mechanics, Royal Institute of Technology (KTH), 100 44 Stockholm, Sweden*

‡*Division of Micro and Nanosystems, Royal Institute of Technology (KTH), 100 44 Stockholm, Sweden*

¶*Södertörn University, 141 89 Stockholm, Sweden*

E-mail: shervin@mech.kth.se

Table of contents

Numerical methods to simulate droplet impact **S2**

Figure S1. Computational domain for the numerical simulations **S2**

References **S5**

Numerical methods to simulate droplet impact

The numerical experiments reported in the main paper for detailed examination of contact line motion near the surface topology are implemented as follows. A two-dimensional representation is chosen for keeping computational cost feasible. The computational domain is shown in Fig. S1. Top and bottom boundaries are treated as solid walls. In addition, we assume that the system is periodic in x direction. Domain and structure size is indicated in Fig. S1.

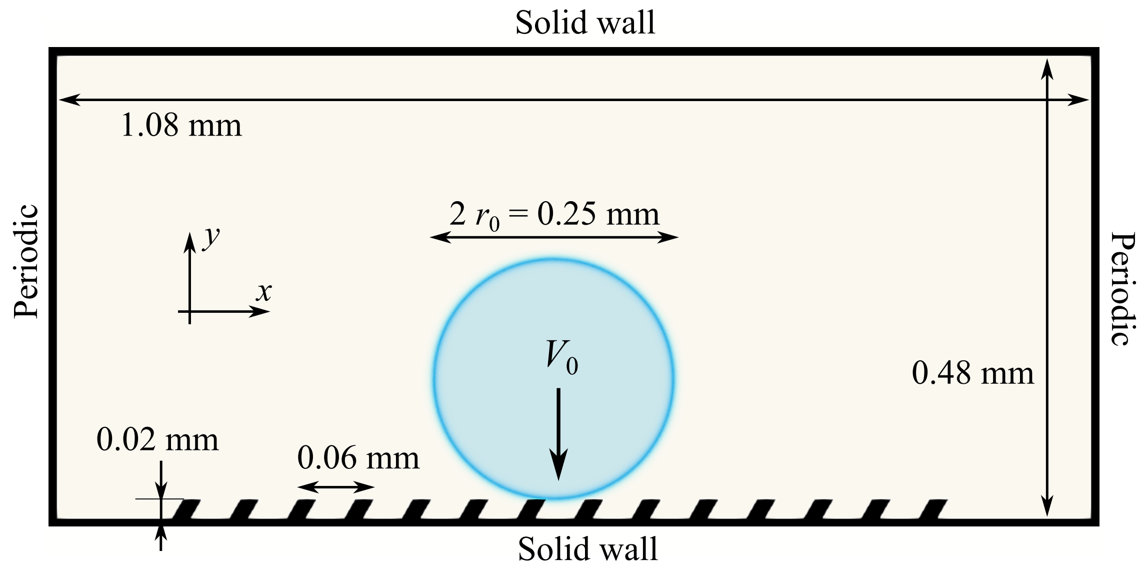

Figure S1: Domain for the numerical simulations. Top and bottom boundaries are solid walls, whereas left and right borders are periodic boundaries. Constant downwards initial velocity V_0 is applied to the whole drop.

We choose the phase-field (PF) model.[S1,S2] The fluid velocity $\vec{u}$ and pressure p are described by the incompressible Navier-Stokes equations. In two dimensional form, these are

$$\rho\,(x,y)\left[\frac{\partial \vec{u}}{\partial t} + (\vec{u}\cdot\nabla)\,\vec{u}\right] = -\nabla p + \nabla\cdot\left[\mu\,(x,y)\left\{\nabla\vec{u} + (\nabla\vec{u})^T\right\}\right] + \phi\,\nabla C, \tag{S1}$$

$$\nabla\cdot\vec{u} = 0. \tag{S2}$$

The $\phi\,\nabla C$ is surface tension force (C and Φ are defined later), $\rho\,(x,y)$ and $\mu\,(x,y)$ are

spatially dependent density and viscosity, respectively. These are expressed as

$$\rho\left(C\right)=\rho_w\frac{C+1}{2}-\rho_a\frac{C-1}{2}\quad\text{and}\quad\mu\left(C\right)=\mu_w\frac{C+1}{2}-\mu_a\frac{C-1}{2},\tag{S3a,b}$$

where $C(x,y)$ is the so called phase function, having value 1 in the water and -1 in the air. The C function is initialised as shown in Fig. S1 to correspond to a water droplet right above the textured surface. Water and air properties are determined from Engineering Tables at temperature 20° Celsius. Water density[S3] is set to $\rho_w = 998.19$ kg/m^3, air density[S4] to $\rho_a = 1.204$ kg/m^3, water viscosity[S5] is chosen as $\mu_w = 1.0005 \times 10^{-3}$ Pa s, air viscosity[S6] as $\mu_w = 1.813 \times 10^{-5}$ Pa s. Velocity and pressure fields are periodic in x direction. No slip ($\vec{u} = 0$) boundary condition is applied on solid walls (Fig. S1). Initial downwards velocity V_0 is applied on droplet, while zero velocity is set everywhere else. The gravity is neglected due to small Bond number of the system $Bo = \rho_w g r_0^2/\sigma = 0.0021$ where $g = 9.8$m/s^2 is the gravitational acceleration.

The evolution of the two phases are governed by the Cahn-Hilliard equations. The first equation is a convection-diffusion equation that governs evolution of C as

$$\frac{\partial C}{\partial t}=\nabla\cdot\left[M\nabla\phi\right]-\vec{u}\cdot\nabla C\,.\tag{S4}$$

Here, M is the phase-field mobility and ϕ is the chemical potential, defined as

$$\phi=\frac{2\sqrt{2}}{3}\frac{\sigma}{\epsilon}\Psi'\left(C\right)-\frac{2\sqrt{2}}{3}\sigma\,\epsilon\nabla^2C.\tag{S5}$$

The chemical potential (S5) has two terms. The first is contribution of the bulk phases, containing derivative of the standard double well potential $\Psi\left(C\right)=\left(C+1\right)^2\left(C-1\right)^2/4$. The second term is contribution of the two phase interface and consequently contains gradient if C function. Pre-factors for each term contain combinations of the surface tension (σ) and the interface thickness (ϵ). The surface tension[S7] we set to $\sigma = 0.0728$ N/m. We treat C

and Φ as periodic in x direction. At solid walls, we enforce zero diffusive flux $\nabla\phi \cdot \hat{n} = 0$ (here, $\hat{n}$ is the normal vector at the boundary) and make use of the wetting condition

$$-\mu_f \epsilon \left(\frac{\partial C}{\partial t} + \vec{u} \cdot \nabla C \right) = \frac{2\sqrt{2}}{3} \sigma\, \epsilon \nabla C \cdot \hat{n} - \sigma \cos\left(\theta_0\right) g'\left(C\right), \tag{S6}$$

where μ_f is the contact line friction, $g\left(C\right) = 0.5 - 0.75C + 0.25C^3$ is a switch function and θ_0 is equilibrium contact angle. If $\mu_f = 0$, condition (S6) enforce θ_0 as the dynamic contact angle. For $\mu_f \neq 0$, dynamic contact angle deviates from θ_0.

To use the PF model in a practical setting, one has to set the constants M (S4), ϵ (S5,S6) and μ_f (S6). This is typically done according to the sharp interface limit[S8] and by calibrating against experiments.[S9] We set $\epsilon = 3.125$ μm and choose $M = 5.486 \times 10^{-9}$ m^4/(N s). To calibrate the line friction parameter μ_f, we follow the previous work[S10,S11] on choosing μ_f for drop spreading experiments. Spreading of a droplet with the initial radius of 0.4 mm on a flat surface is experimentally observed with a high-speed camera at a frame rate of 52000 s^{-1} and the spreading radius and the spreading time are recorded. Experiments of a droplet spreading on a flat surface are modelled numerically to determine the line friction parameter. The Cahn-Hilliard Navier-Stokes equations are solved in cylindrical coordinates using in-house software "FemLego" to obtain the spreading radius for different values of μ_f. FemLego is an adaptive finite element toolbox where weak formulation of partial differential equations is defined on a MAPLE worksheet.[S12] The spreading curves are fitted in order to estimate the best-fit line friction parameter. The friction parameter was identified to be μ_f = 0.08 Pa s. Further details can be found in Ref. S10,S11.

After calibration, the drop impact simulations are carried out. These simulations are done using the open-source finite element solver FreeFEM.[S13] Equations and boundary conditions (S1–S6) are linearised, rendered dimensionless, written in the weak form and inputted into FreeFEM. Mesh resolution of $\Delta s_1 = 10.42$ μm away from the interface and $\Delta s_2 = 0.69$ μm close to the interface was used. Constant time step of $\Delta t = 0.0309$ μs was used. The

unstructured finite element mesh was adapted to C function each 50 time steps. FreeFEM version 4.6 was used to produce the results shown in the main paper. Scripts to reproduce the simulation are available in Ref. S14. Video animation of the impact simulation is available as Supplemental Material. The impact speed in the supporting video is 2 m/s.

References

[S1] Jacqmin, D. Contact-line dynamics of a diffuse fluid interface. *J. Fluid Mech.* **2000**, *402*, 57–88.

[S2] Carlson, A. Capillarity and dynamic wetting. Ph.D. thesis, KTH Royal Institute of Technology, 2012.

[S3] Engineering ToolBox, Water – Density, Specific Weight and Thermal Expansion Coefficients. 2003; https://www.engineeringtoolbox.com/water-density-specific-weight-d_595.html, [online, Accessed 5th of November, 2021].

[S4] Engineering ToolBox, Air – Density, Specific Weight and Thermal Expansion Coefficient vs. Temperature and Pressure. 2003; https://www.engineeringtoolbox.com/air-density-specific-weight-d_600.html, [online, Accessed 5th of November, 2021].

[S5] Engineering ToolBox, Water - Dynamic (Absolute) and Kinematic Viscosity vs. Temperature and Pressure. 2004; https://www.engineeringtoolbox.com/water-dynamic-kinematic-viscosity-d_596.html, [online, Accessed 5th of November, 2021].

[S6] Engineering ToolBox, Air - Dynamic and Kinematic Viscosity. 2003; https://www.engineeringtoolbox.com/air-absolute-kinematic-viscosity-d_601.html, [online, Accessed 5th of November, 2021].

[S7] Engineering ToolBox, Surface Tension of Water in contact with Air. 2004; https://www.engineeringtoolbox.com/water-surface-tension-d_597.html, [online, Accessed 5th of November, 2021].

[S8] Yue, P.; Zhou, C.; Feng, J. J. Sharp-interface limit of the Cahn–Hilliard model for moving contact lines. *J. Fluid Mech.* **2010**, *645*, 279–294.

[S9] Yue, P.; Feng, J. J. Wall energy relaxation in the Cahn-Hilliard model for moving contact lines. *Phys. Fluids* **2011**, *23*, 012106.

[S10] Carlson, A.; Bellani, G.; Amberg, G. Universality in dynamic wetting dominated by contact-line friction. *Phys. Rev. E* **2012**, *85*, 045302(R).

[S11] Yada, S.; Allais, B.; van der Wijngaart, W.; Lundell, F.; Amberg, G.; Bagheri, S. Droplet Impact on Surfaces with Asymmetric Microscopic Features. *Langmuir* **2021**, *37*, 10849–10858.

[S12] Amberg, G.; Tönhardt, R.; Winkler, C. Finite element simulations using symbolic computing. *Math. Comp Simul.* **1999**, *49*, 257 – 274.

[S13] Hecht, F. New development in FreeFem++. *J. Numer. Math.* **2012**, *20*, 251–265.

[S14] Lācis, U.; Bagheri, S. https://github.com/UgisL/FreeFEM-NS-CH. 2020–2022.
